# Respiratory Outcomes in Patients Following COVID-19-Related Hospitalization: A Meta-Analysis

**DOI:** 10.3389/fmolb.2021.750558

**Published:** 2021-10-06

**Authors:** Tao Guo, Fangfang Jiang, Yufei Liu, Yunpeng Zhao, Yiran Li, Yihua Wang

**Affiliations:** ^1^ Biological Sciences, Faculty of Environmental and Life Sciences, University of Southampton, Southampton, United Kingdom; ^2^ Department of Mathematical Sciences, Faculty of Social Sciences, University of Southampton, Southampton, United Kingdom; ^3^ School of Food Science and Technology, Jiangnan University, Wuxi, China; ^4^ School of Medicine and Integrated Medicine, Nanjing University of Chinese Medicine, Nanjing, China; ^5^ School of Artificial Intelligence and Information Technology, Nanjing University of Chinese Medicine, Nanjing, China

**Keywords:** COVID-19, follow-up, pulmonary function test, FVC, DLCO, synthesis review, meta-analysis

## Abstract

**Background:** To determine the respiratory outcomes in patients following COVID-19-related hospitalization.

**Methods:** Systematic review and meta-analysis of the literature.

**Results:** Forced vital capacity (FVC, % of predicted): 0–3 months post discharge: 96.1, 95% CI [82.1–110.0]; 3–6 months post discharge: 99.9, 95% CI [84.8, 115.0]; >6 months post discharge: 97.4, 95% CI [76.8–118.0]. Diffusing capacity of the lungs for carbon monoxide (DLCO, % of predicted): 0–3 months post discharge: 83.9, 95% CI [68.9–98.9]; 3–6 months post discharge: 91.2, 95% CI [74.8–107.7]; >6 months post discharge: 97.3, 95% CI [76.7–117.9]. Percentage of patients with FVC less than 80% of predicted: 0–3 months post discharge: 10%, 95% CI [6–14%]; 3–6 months post discharge: 10%, 95% CI [2–18%]; >6 months post discharge: 13%, 95% CI [8–18%]. Percentage of patients with DLCO less than 80% of predicted: 0–3 months post discharge: 48%, 95% CI [41–56%]; 3–6 months post discharge: 33%, 95% CI [23–44%]; >6 months post discharge: 43%, 95% CI [22–65%].

**Conclusion:** The meta-analysis confirms a high prevalence of persistent lung diffusion impairment in patients following COVID-19-related hospitalization. Routine respiratory follow-up is thus strongly recommended.

## Introduction

To date, over 200 million people worldwide have recovered from COVID-19 (https://www.worldometers.info/coronavirus/) (Worldometers (2020). Worl, 2020), but concern remains that some organs, including the lungs, might suffer long-term impairment following recovery from acute infections. Individual studies have shown that residual abnormalities of pulmonary function were observed in a subgroup of recovered COVID-19 patients, with the most common finding being a reduction in gas transfer as measured by diffusing capacity of the lungs for carbon monoxide (DLCO) (Hull et al., 2020; Dhawan et al., 2021; Thomas et al., 2021). In this study, with meta-analysis, we aimed to determine the short (0–3 months), medium (3–6 months) and long (>6 months) respiratory outcomes in patients following COVID-19-related hospitalisation. The findings will instruct appropriate interventions for subsequent increased healthcare utilisation post-COVID-19.

## Method

### Criteria for Inclusion

We included randomised controlled trials (RCTs) and observational studies (cross-sectional, longitudinal, case-control and cohort) of patients with a confirmed diagnosis of COVID-19. The studies included aimed to determine the respiratory outcomes, in particular forced vital capacity (FVC) and diffusing capacity of the lungs for carbon monoxide (DLCO), in patients following COVID-19-related hospitalisation. The selected studies had to follow the ATS / ERS clinical guidelines. The included literatures should be published before May 15, 2021.

### Criteria for Exclusion

Study’s subjects who were not infected with COVID-19. Studies didn’t report the time of hospital discharge or the time was calculated from diagnosis of COVID-19. Studies did not report FVC (% of predicted) or DLCO (% of predicted) or FVC <80% of predicted or DLCO <80% of predicted. Animal experiments, medical records, case reports, famous medical experience and review were excluded.

### Literature Retrieval and Selection

Firstly, according to the literature inclusion criteria, two researchers independently searched at Pubmed, ScienceDirect, Embase and Web of Science. Secondly, two researchers selected the literature and extracted the data independently in accordance with the standard data extraction table. When it came to divergences, a third researcher did the judgement. After the discussion, researchers reached a consensus. Finally, after the extraction and input of the data, two independent researchers did the subsequent analysis.

### Extraction of Data

According to the inclusion criteria, we assessed the design of research, patients, and outcome indicators. First author, published year, number of cases, nationality, ages, body mass index (BMI), smoking status, respiratory comorbidities, time of assessment and, index quantity of FVC, % of predicted, DLCO, % of predicted; FVC <80% of predicted and DLCO <80% of predicted were extracted from eligible studies.

### Quality Assessment of Articles

The studies with randomised controlled trials were evaluated by Newcastle-Ottawa Scale (Bellan et al., 2021). As for no controlled trials, it includes the following aspects: 1) selection: Representativeness of the exposed cohort, selection of the non-exposed cohort, Ascertainment of exposure, Demonstration that outcome of interest was not present at start of study; 2) comparability: Research control matched important factors, but also controlled other important factors; and 3) outcome: assessment of outcome, follow-up long enough for outcomes to occur, adequacy of follow up of cohorts.

### Synthesis and Analysis of Data

We used package “meta (version 4.18-0)” in R 4.0.1 and R studio to perform meta-analysis of the following pulmonary function tests (PFTs) indexes (1. FVC, % of predicted; 2. DLCO, % of predicted; 3. FVC <80% of predicted; 4. DLCO <80% of predicted.). Patients were divided into three groups: less than 3 months (0–3 months), more than or equal to 3 months and less than 6 months (3–6 months), and more than or equal to 6 months (≥6 months). We re-calculated the median (first quantile, third quantile) to mean ± standard deviation (SD) for FVC (% of predicted) and DLCO (% of predicted) in several studies. Statistical heterogeneity was measured through the I^2^ statistic and classified as low (I^2^ < 25%), moderate (I^2^ 25–50%), and high (I^2^ > 50%) (Melsen et al., 2014). Subgroup analysis, according to the outcome assessment and severity, was performed. Sensitivity analysis was also employed to assess the change in pooled prevalence due to the selective exclusion of studies.

## Results

### Literature Extraction

**FIGURE 1:**
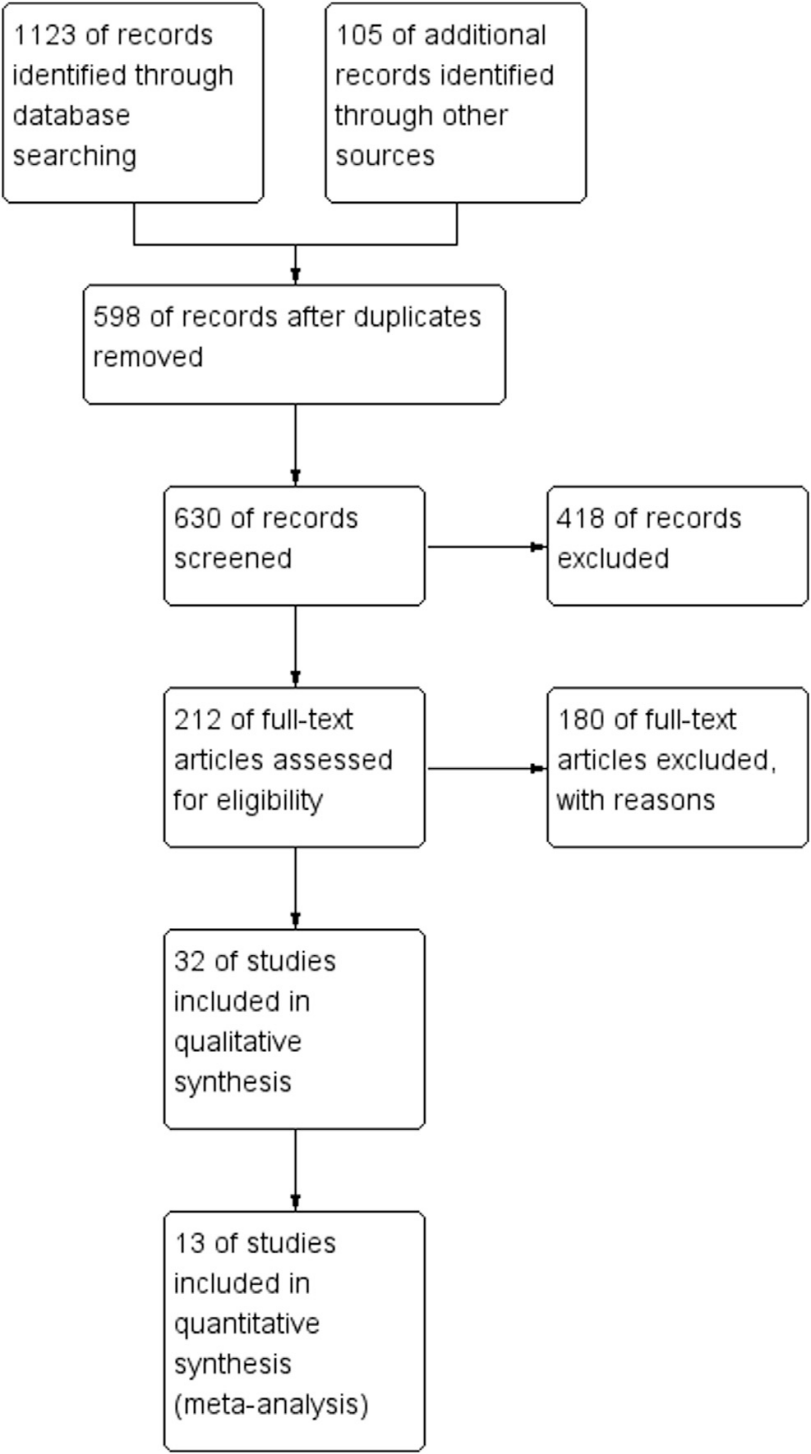
Workflow showing literature extraction (details provided in Methods).

A total of 1,123 articles was retrieved from databases via the retrieval methods. Duplicate literatures were excluded through titles and abstracts. By reading the full text, we excluded 1,110 papers and conference abstracts with incomplete or no specific research method. Finally, 13 papers published in English were included (Liang et al., 2020; Huang et al., 2020; Venturelli et al., 2021; You et al., 2020; Lerum et al., 2021; Daher et al., 2020; Wu et al., 2021; Bellan et al., 2021; Li et al., 2020; van den Borst et al., 2020; Mo et al., 2020; Zhao et al., 2020; Huang et al., 2021), with a total of 3,455 patients. The evaluation of the quality of included studies by Newcastle-Ottawa Scale (NOS) (Stang et al., 2018) showed that two studies had a poor quality and the rest 11 studies passed the quality control. The basic characteristics of the included literatures were detailed in Table 1 and the procedure of literature retrieval and selection was shown in Figure 1.

**TABLE 1:** Basic characteristics of included studies.

Author	Country	Design	Participants male/female	Age (years)	BMI (kg/m2)	Smoking	Respiratory comorbidities	Time of assessment	Quality rating
Huang et al., (2020)	China	retrospective	57 26M/31F	46.7 ± 13.7	23.9 ± 3.5	History of smoking 9 (15.7%)	No patient was reported having chronic repiratory diseases	30 days after discharge from the hospital	high
Venturelli et al., (2021)	Italy	prospective	767 515M/252F	63 ± 13.6	NR	Active smoker 33 (4.3%) History of smoking 179 (23.3%)	NR	81 (66–106) days after hospital discharge	high
You et al., (2020)	China	prospective	18 10M/8F	50.7 ± 12.1	26.4 ± 2.8	NR	No patient was reported having chronic repiratory diseases	38 ± 13.4 days after hospital discharge	high
Lerum et al., (2021)	Norway	prospective	103 54M/49F	59 (49–72)	25.8 (23.8–29.6)	Current smoker 3 (3.4%) previous smoker 34 (39%)	NR	3 months after hospital admission	poor
Daher et al., (2020)	Germany	prospective	33 22M/11F	64 ± 3	28 (24–31)	NR	7 (21%)	6 weeks after hospital discharge	high
Wu et al., (2021)	China	prospective, longitudinal, cohort	83 47M/36F	60 (52–66)	25 (23.5–27.1)	NR	No patient was reported having chronic repiratory diseases	3 months, 6 months, 9 months, 12 months after hospital discharge	high
Liang et al., (2020)	China	Prospective	76 21M/55F	41.3 ± 13.8	23.7 ± 4.5	NR	Cough 45 (60%) Increased sputum production 33 (43%) Activity chest tightness and palpitations 47 (62%)	3 -months follow-up study after discharge	high
Bellan et al., (2021)	Italy	prospective cohort study	238 142M/96F	61 (51–71)	NR	Never 139(58.4%)Former 74(31.1%)Current 25(10.5%)Pack-years,median(IQR) 15(7.25–36)	No patient was reported having chronic repiratory diseases	4 months after discharge	high
Li et al., (2020)	China	a prospective study	18	NR	NR	History of smoking 3(16.6%)	history of tuberculosis 1 (5.5%)	Near discharge and in quarantine period (2 weeks after discharge)	high
van den Borst et al., (2020)	Netherlands	Prospective	124 74M/50F	59 ± 14	NR	Never 48(39%)Former 74(60%)Current 2(2%)	asthma 12 (10%) chronic lung diseases 23 (19%) other lung diseases 4 (3%)	Three months after recovery	high
Mo et al., (2020)	China	Prospective	110 55M/55F	49.1 ± 14.0	23.5 ± 3.0	History of smoking 13 (11.8%)	asthma 1 (0.9%) chronic bronchitis 1 (0.9%) bronchiectasis 1 (0.9%)	At time of hospital discharge	poor
Zhao et al., (2020)	China	retrospective	55 22M/23F	47.7 ± 15.5	NR	active 2 (3.6%) former 2 (3.6%)	cough 7 (43.75%)	3 months after hospital discharge	high
Huang et al., (2021)	China	prospective cohort study	1733 897M/836F	57 (47–65)	NR	Never-smoker 1585/1731 (92%) Current smoker 102/1731 (6%) Former smoker 44/1731 (3%)	Chronic obstructive pulmonary disorder 31 (2%)	153.0 (146.0–160.0) days after hospital discharge	high

NR, not reported; BMI, body mass index; M, male; F, female.

Among the included studies, 10 studies reported FVC (% of predicted), eight studies reported DLCO (% of predicted), six reported FVC <80% of predicted, and nine reported DLCO <80% of predicted. Wu et al. (2021) *.* reported all the indexes of the patients after the 3, 6 and 12 months following COVID-19-related hospitalisation (Table 2). For those data reported in the form of median (first quantile, third quantile), we used R studio to re-calculate them into mean ± SD (Table 3).

**TABLE 2:** Summary of studies included pulmonary function test.

	Wu et al., (*n* = 83)			You et al., (*n* = 18)	Zhao et al., (*n* = 55)
FVC, % of predicted	92 (81–99)	94 (85–104)	98 (89–109)	105.1 ± 23.3	NR
DLCO, % of predicted	77 (67–87)	76 (68–90)	88 (78–101)	NR	NR
FVC, < 80% of predicted	19	13	9	3	NR
DLCO, < 80% of predicted	46	45	27	NR	9
Time of assessment	3 months	6 months	12 months	38 ± 13.4 days after hospital discharge	3 months after hospital discharge

	Lerum et al., (*n* = 103)	Borst et al., (*n* = 124)	Li et al., (*n* = 18)	Daher, A et al. (*n* = 33)	Venturelli, S et al. (*n* = 767)
FVC, % of predicted	94 (76–121)	NR	91.5 ± 17.3	NR	95(84–106), *f*
DLCO, % of predicted	83 (72–92)	81 ± 17	NR	65(53–73)	96(81–112), *p*
FVC, < 80% of predicted	7	NR	NR	NR	NR
DLCO, < 80% of predicted	24	41	NR	NR	NR
Time of assessment	3 months after hospital discharge	3 months after recovery	Near to discharge and 2 weeks after	56 days from discharge to follow-up	80(median)days after discharge

	Huang et al. (*n* = 349)	Bellan et al., (*n* = 224)	Liang et al., (*n* = 76)	Huang et al., (*n* = 57)	Mo et al., (*n* = 110)
FVC, % of predicted	NR	98.5 (90–109)	107.1 ± 12.3	100.96 ± 15.93	93.59 ± 12.25
DLCO, % of predicted	NR	79 (69–89), *q*	NR	78.38 ± 13.59	78.18 ± 14.29
FVC, < 80% of predicted	14	NR	NR	6	10
DLCO, < 80% of predicted	114, *I*	113, *q*	15	30	51
Time of assessment	153.0 (146.0–160.0) days after hospital discharge	4 months after hospital discharge	3 months after hospital discharge	1 month after hospital discharge	when discharged from hospital

*f: n = 717, p: n = 680, q: n = 219, l: n = 334.*

NR, not reported; FVC, forced vital capacity; DLCO, diffusing capacity for carbon monoxide.

**TABLE 3:** Summary of re-calculations of median into mean using R studio.

Author	Time	FVC.mean	FVC.sd	FVC.n	DLCO.mean	DLCO.sd	DLCO.n
Frija-Masson	30 days after symptoms onset	91.7	11.14	50	91.27	11.23	50
Daher, A	56 days from discharge to follow-up	NR	NR	NR	88.93	17.67	33
Venturelli, S	80(median)days after discharge	95.02	15.99	717	95.48	16.6	680
Lerum	3 months after hospital discharge	102.1	37.78	103	99.68	34.9	103
Darley,D.R	113(median)days after diagnosis	106.91	15.07	65	106.88	14.79	65
Belan	4 months after hospital discharge	99.9	14.3	224	99.79	14.28	219
Wu	3 months	89.11	14.73	83	88.45	14.13	83
Wu	6 months	95.07	14.3	83	95.26	14.26	83
Wu	12 months	100.19	15.53	83	99.67	15.56	83

FVC, forced vital capacity; DLCO, diffusing capacity for carbon monoxide; NR, Not reported.

**FIGURE 2:**
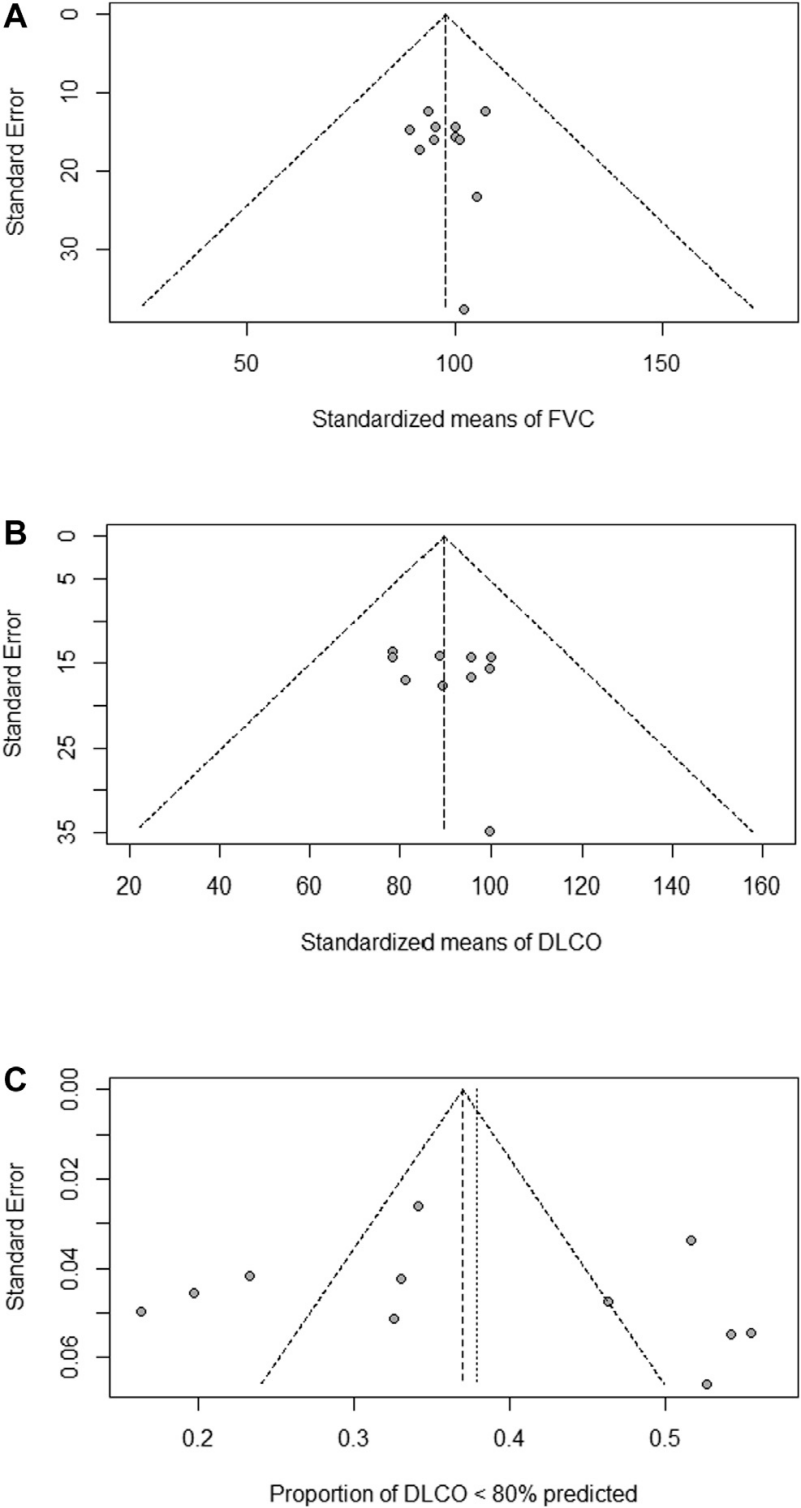
No publication bias of the meta-analysis. Funnel plots of FVC (% of predicted) **(A)**, DLCO (% of predicted) **(B)** and DLCO <80% of predicted **(C)** were shown. Each dot represents a study.

Publication bias refers to the fact that research results with statistical significance are more likely to be reported and published than those without statistical significance and invalid results (DeVito and Goldacre, 2019). We examined the publication bias of meta-analysis of each indicator. There was no publication bias in FVC (% of predicted; *p* = 0.93; Figure 2A), DLCO (% of predicted; *p* = 0.54; Figure 2B) and DLCO (<80% of predicted; *p* = 0.94; Figure 2C). For FVC <80% of predicted, less than 10 studies were included, so publication bias was not tested.

### Comparison of Longitudinal Changes in FVC (% of Predicted)

**FIGURE 3:**
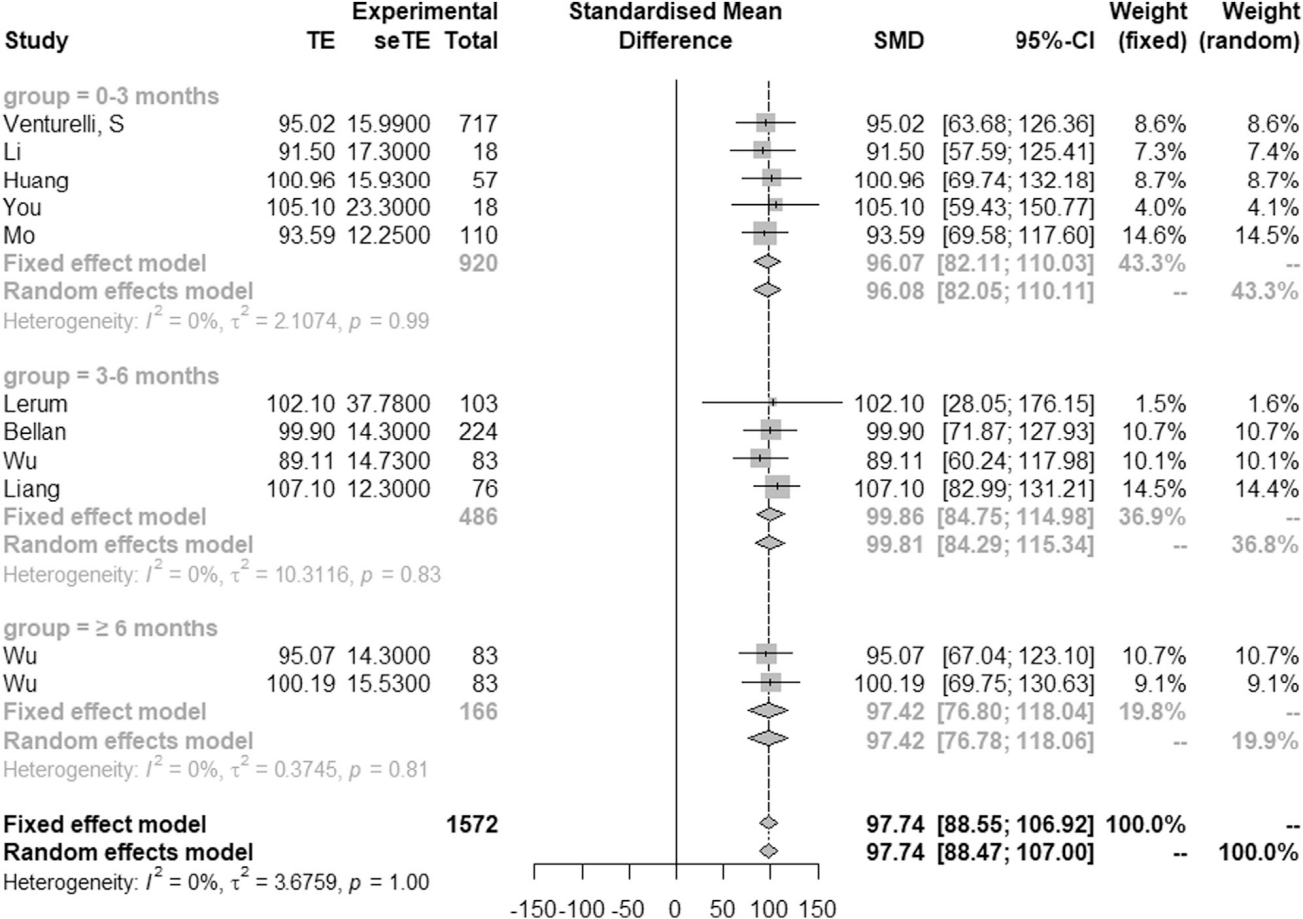
Comparison of longitudinal changes in FVC (% of predicted). Forest plot showing meta-analysis of FVC (% of predicted).

Nine studies with 11 groups of data showed the results of FVC (% of predicted). Based on the time of patients discharged from hospital, we divided them into three groups: 0–3 months, 3–6 months and ≥6 months. FVC (% of predicted) in 0–3 months, 3–6 months and ≥6 months post discharge were 96.1 (95% CI [82.1–110.0]), 99.9 (95% CI [84.8–115.0]) and 97.4 (95% CI [76.8–118.0]), respectively. In this study, heterogeneity was extremely low (I^2^ = 0%), and the overall value of FVC (% of predicted) in all studies was 97.7 (95% CI [88.6–106.9]) (Figure 3).

### Comparison of Longitudinal Changes in DLCO (% of Predicted)

**FIGURE 4:**
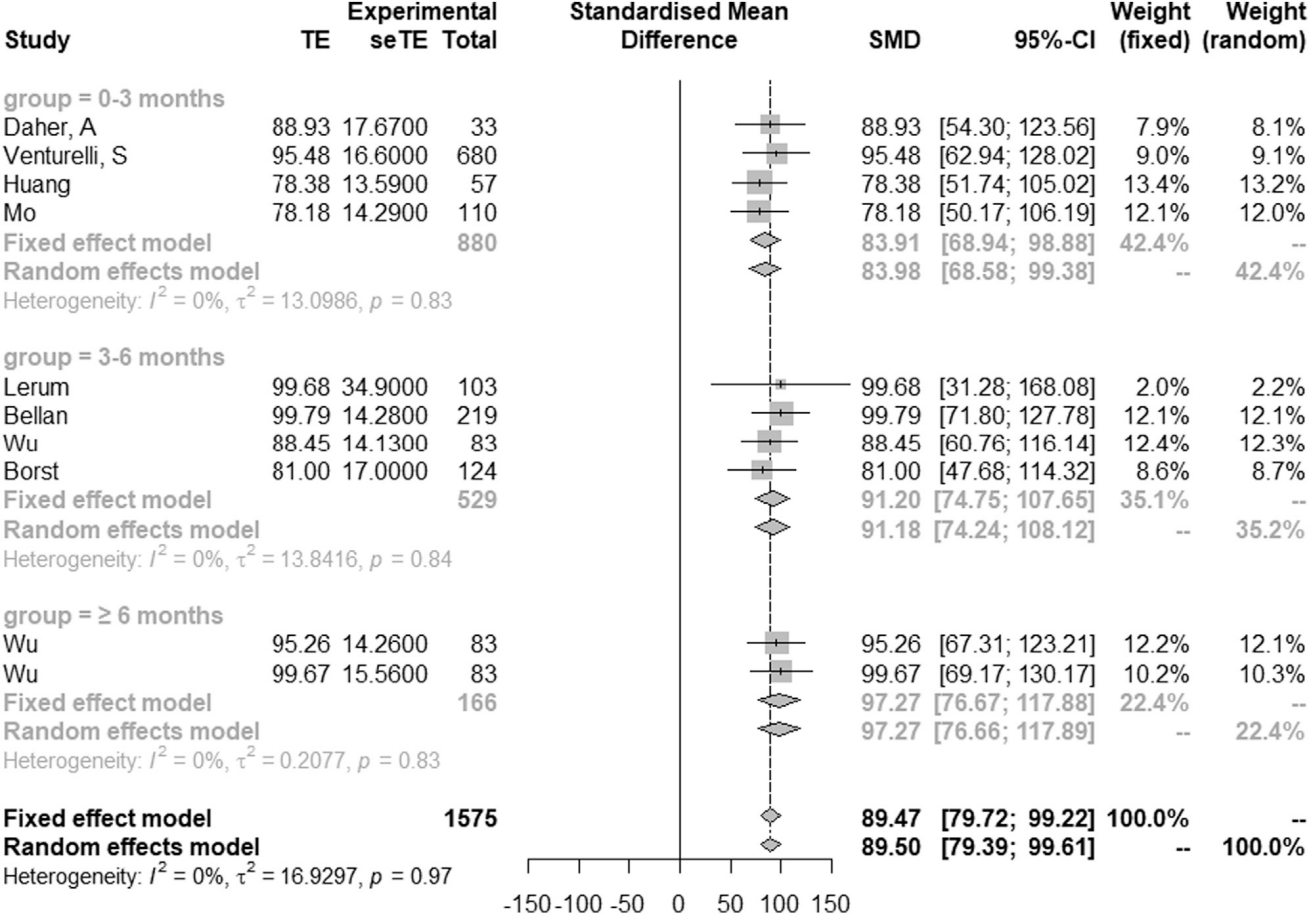
Comparison of longitudinal changes in DLCO (% of predicted). Forest plot showing meta-analysis of DLCO (% of predicted).

Eight studies with 10 groups of data showed the results of DLCO (% of predicted). DLCO (% of predicted) in 0–3 months, 3–6 months and ≥6 months post discharge were 83.9 (95% CI [68.9–98.9]), 91.2 (95% CI [74.8–107.7]) and 97.3 (95% CI [76.7–117.9]), respectively. Heterogeneity was considered low (I^2^ = 0%) using a fixed effect model (Melsen et al., 2014; Bellou et al., 2016) (Figure 4).

Comparison of longitudinal changes in the percentage of patients with FVC <80% of predicted.

**FIGURE 5:**
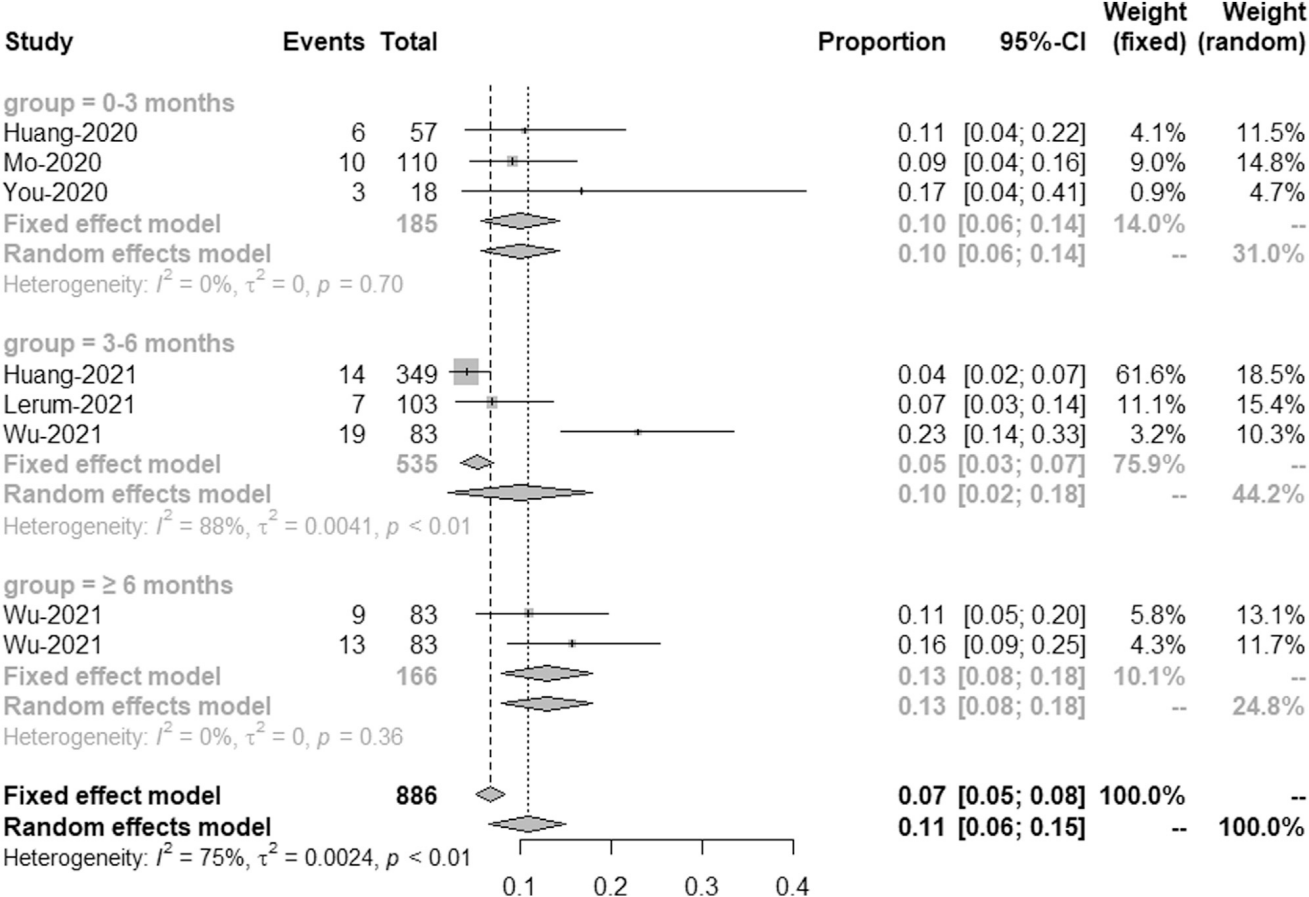
Comparison of longitudinal changes in the percentage of patients with FVC <80% of predicted. Forest plot showing meta-analysis of the percentage of patients with FVC <80% of predicted.

These included six studies, which in total have eight groups of data showed the percentage of patients with FVC less than 80% of predicted. Based on the time of patients being discharged from hospital, we divided them into three groups: 0–3 months, 3–6 months and greater than 6 months. Meta-analysis showed that the percentage of patients with FVC less than 80% of predicted in 0–3 months, 3–6 months and ≥6 months post discharge was 10% (95% CI [6–14%]), 10% (95% CI [2–18%]) and 13% (95% CI [8–18%], respectively. The heterogeneity of 3–6 months was large, so the sensitivity analysis was carried out in this study. We removed the study from Wu *et al.* and got the meta-analysis result of this subgroup, which was 4% (95% CI [3–6%]) with I^2^ = 6% (Figure 5).

Comparison of longitudinal changes in the percentage of patients with DLCO <80% of predicted.

**FIGURE 6:**
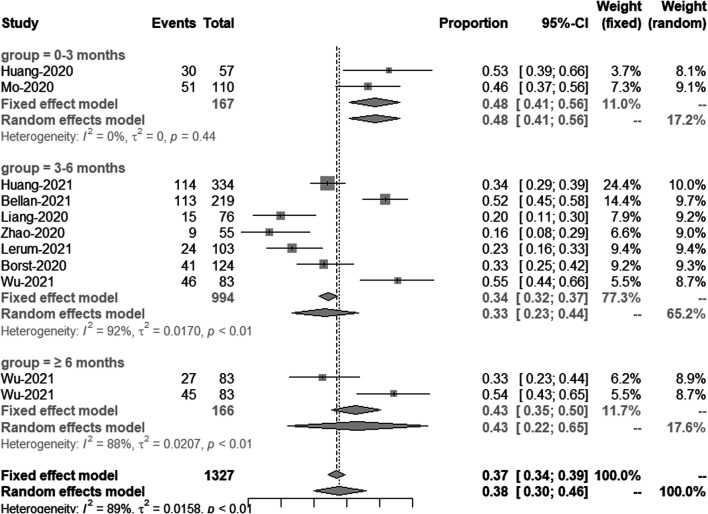
Comparison of longitudinal changes in the percentage of patients with DLCO <80% of predicted. Forest plot showing meta-analysis of the percentage of patients with DLCO <80% of predicted.

This included nine studies, which have 11 groups of data shows the results of DLCO less than 80% of predicted. Meta-analysis showed a significant and persistent reduction in DLCO over the study period. The percentage of patients with DLCO less than 80% of predicted in 0–3 months, 3–6 months and ≥6 months post discharge was 48% (95% CI [41–56%]), 33% (95% CI [23–44%]) and 43% (95% CI [22–65%]), respectively (Figure 6)**.**

## Discussion

Post-acute COVID-19 syndrome, also known as long COVID, encompasses a wide range of physical and mental health symptoms that persist after recovery from acute SARS-CoV-2 infections (Nalbandian et al., 2021). Systematic studies of sequelae after recovery from acute COVID-19 are demanded to inform effective clinical management for patients suffered from long COVID.

We recently reported the 3 months, 6 months, 9 months, and 12 months respiratory outcomes in patients following COVID-19-related hospitalisation from a relatively small prospective cohort (*n* = 83) (Wu et al., 2021). In this study, we conducted meta-analysis to determine the short (0–3 months), medium (3–6 months) and long (>6 months) respiratory outcomes in patients following COVID-19-related hospitalisation. Significantly, we found a persistent reduction in DLCO over the study period, consistent with earlier reports (E et al., 2021). Low DLCO could be caused by interstitial changes or pulmonary vascular abnormalities following COVID-19 infections (Lang et al., 2020; Patel et al., 2020; Hanidziar and Robson, 2021). Our study has shown that up to a third of COVID patients still have evidence of defect DLCO 1 year after discharge (Wu et al., 2021), although longer term follow-up with a larger cohort will be required to confirm this observation.

In general, the heterogeneity of the studies included in the meta-analysis was low. However, the heterogeneity of DLCO less than 80% of predicted was higher, which may be caused by different ethnic groups, ages, disease severity, therapies and other factors. In general, the models we used were robust and reliable.

There are several limitations in this study. Firstly, age, sex ratio, nationality and disease severity of the patients included in the study are quite different, which may cause great heterogeneity and affect the final research results. Secondly, we only selected four indicators of lung function, so we cannot investigate the relationship between other indicators and discharge time. To be consistent and comparable with our earlier publication (Wu et al., 2021), we excluded those studies without data on FVC and/or DLCO values <80% of predicted. This might cause some false positive results considering the mean age of included patients is over 50 (van den Borst et al., 2020; Barisione and Brusasco, 2021; Milanese et al., 2021). In addition, pre-existing comorbidities for most COVID-19 patients are not known, which might cause certain bias of the results. Despite of these limitations, our findings in this meta-analysis are consistent with our previous report (Wu et al., 2021), confirming a high prevalence of persistent lung diffusion impairment in patients following COVID-19-related hospitalisation. Routine respiratory follow-up is thus strongly recommended.

## Data Availability

The original contributions presented in the study are included in the article/Supplementary Material, further inquiries can be directed to the corresponding author.

## Abbreviations

BMI, body mass index; COVID-19, Corona virus disease 2019; DLCO, diffusing capacity for carbon monoxide; FVC, forced vital capacity; NR, not reported; PFTs, pulmonary function tests.
